# Supplementary material for: Targeted malaria elimination interventions reduce Plasmodium falciparum infections up to 3 kilometers away
Source: medRxiv. 2023 Nov 30:2023.09.19.23295806. Originally published 2023 Sep 20. Preprint. [Version 2] doi: 10.1101/2023.09.19.23295806 (PMC10543053; doi:10.1101/2023.09.19.23295806)
Supplement: Supplement 1 [file NIHPP2023.09.19.23295806v2-supplement-1.pdf]

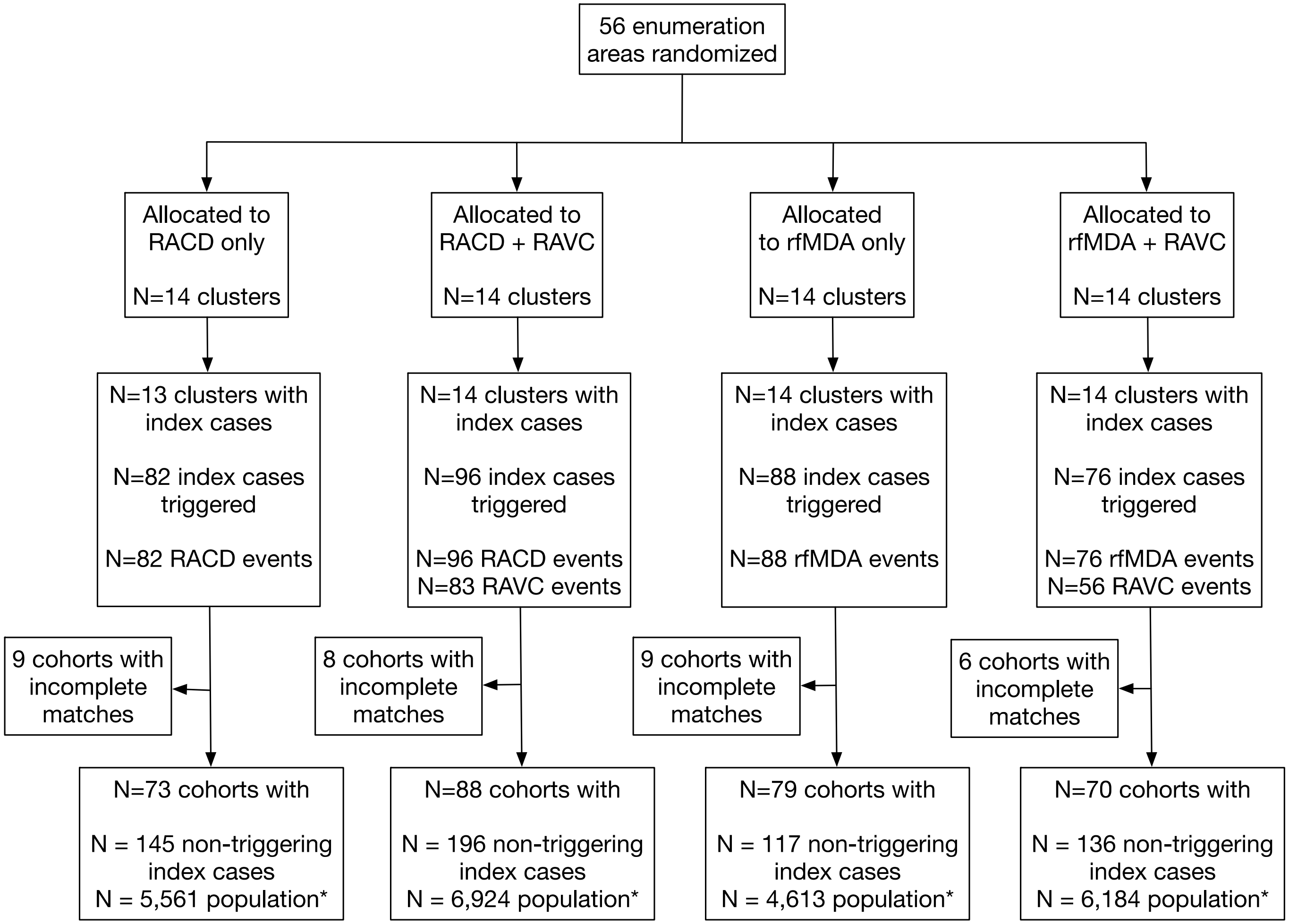

* Some individuals close to cluster boundaries contributed to cohorts in more than one study arm.

Figure S1. Diagram of study randomization, index cases, and population by arm
RACD: reactive case detection. rfMDA: reactive, focal mass drug administration. RAVC: reactive vector control.

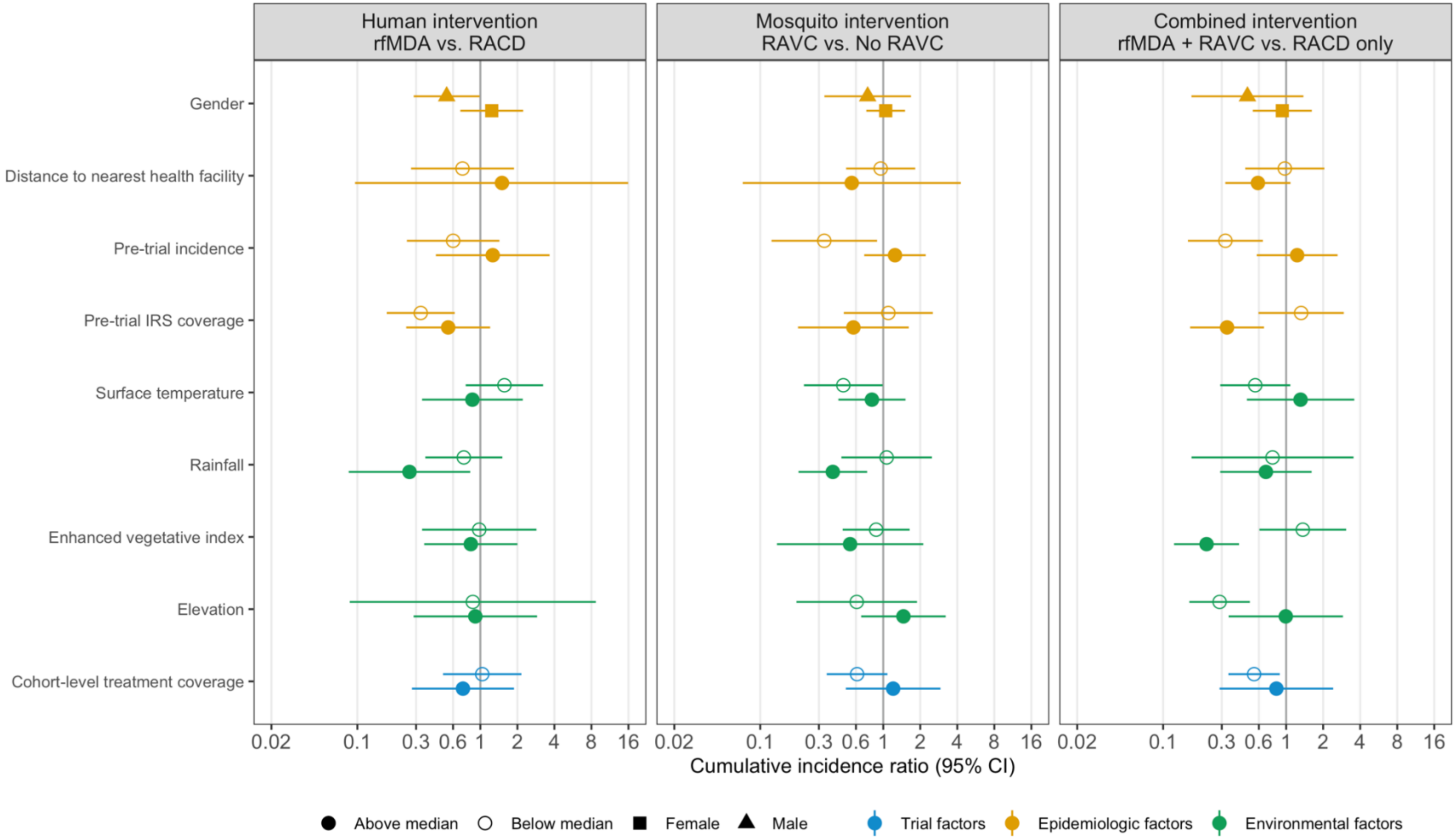

Figure S2. Spillover effect estimates on cumulative incidence within subgroups
Cumulative incidence ratios estimated with hierarchical TMLE; outcome models were fit with cohort-level data. Models were adjusted for covariates that were screened separately for each model using a likelihood ratio test. Models for rfMDA + RAVC vs. RACD were unadjusted due to data sparsity. Confidence intervals account for cohort overlap. For rfMDA and RACD arms, the analysis includes the period from 0-35 days following index case detection for direct effects and 21-56 days for spillover effects. For rfMDA+RAVC and RAVC only arms, the analysis includes

the period from 0-6 months following index case detection for direct effects and 17 days to 6 months for spillover effects. Total effects analyses include the person-time for the direct effects and spillover effects analyses. Direct effect includes treated in target zone. Spillover effect includes intervention non-recipients up to 1km from an index case. Total effect includes all individuals (intervention recipients and non-recipients) up to 1km from index case. For the human intervention, confidence interval upper bounds were truncated at 16 for above median distance to the nearest health facility (observed value: 23).

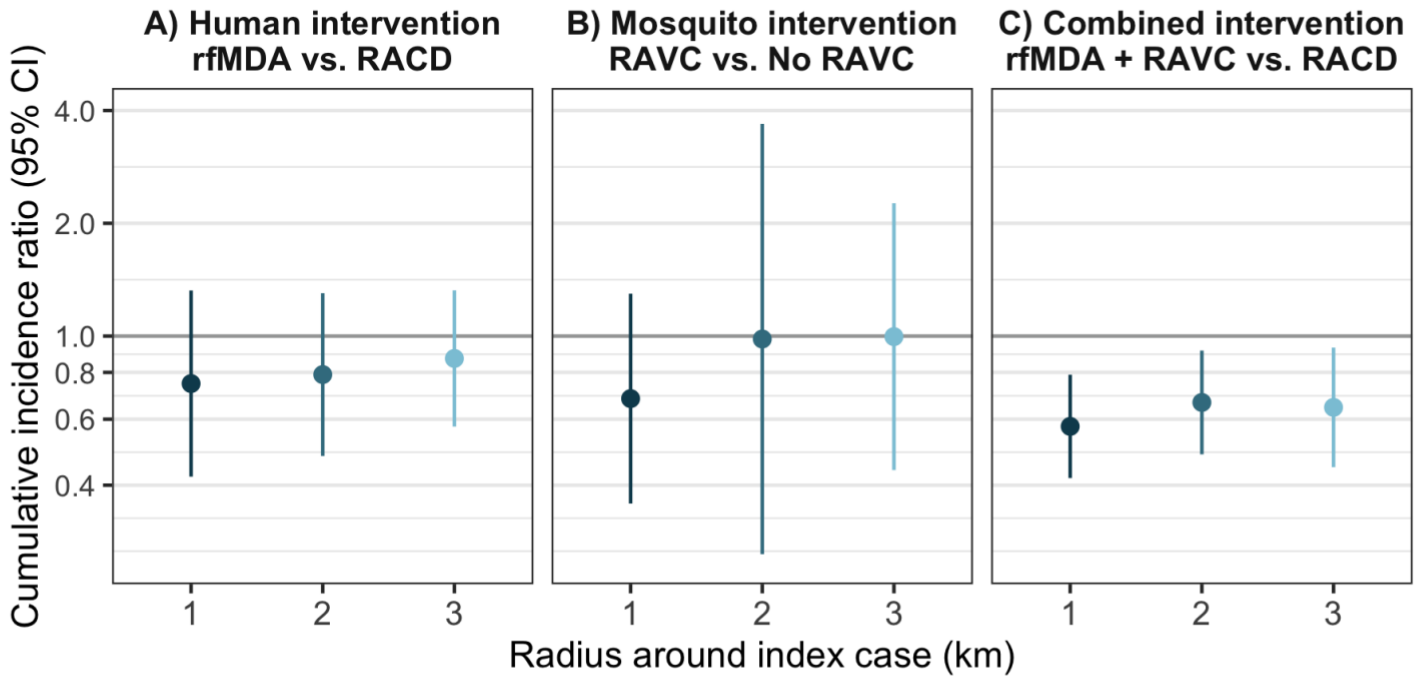

Figure S3. Sensitivity analyses for spillover effects on cumulative incidence of malaria with different distance radii
For rfMDA and RACD arms, the primary analysis includes the period from 0-35 days following index case detection for direct effects and 21-56 days for spillover effects; the alternative observation period analysis includes the period from 0-21 days following index case detection for direct effects and 21 to 42 days for spillover effects. For rfMDA+RAVC and RAVC only arms, the primary analysis includes the period from 0-6 months following index case detection for direct effects and 17 days to 6 months for spillover effects; the alternative observation period analysis includes the period from 0-7 days following index case detection for direct effects and 17 to 90 days for spillover effects. Total effects analyses include the person-time for the direct effects and spillover effects analyses. Direct effect includes intervention recipients in target zone. Spillover effect includes intervention non-recipients up to 1km from an index case in the primary analysis and up to 2km or 3km in sensitivity analyses. Total effect includes all individuals (intervention recipients and non-recipients) up to 1km from index case in the primary analysis and up to 2km or 3km in sensitivity analyses. Includes cohort-level analyses for all estimates except spillover effects of the combined intervention. All incidence outcome models were fit with cohort-level data except for models of spillover effects of rfMDA vs. RACD and rfMDA + RAVC vs. RACD only.

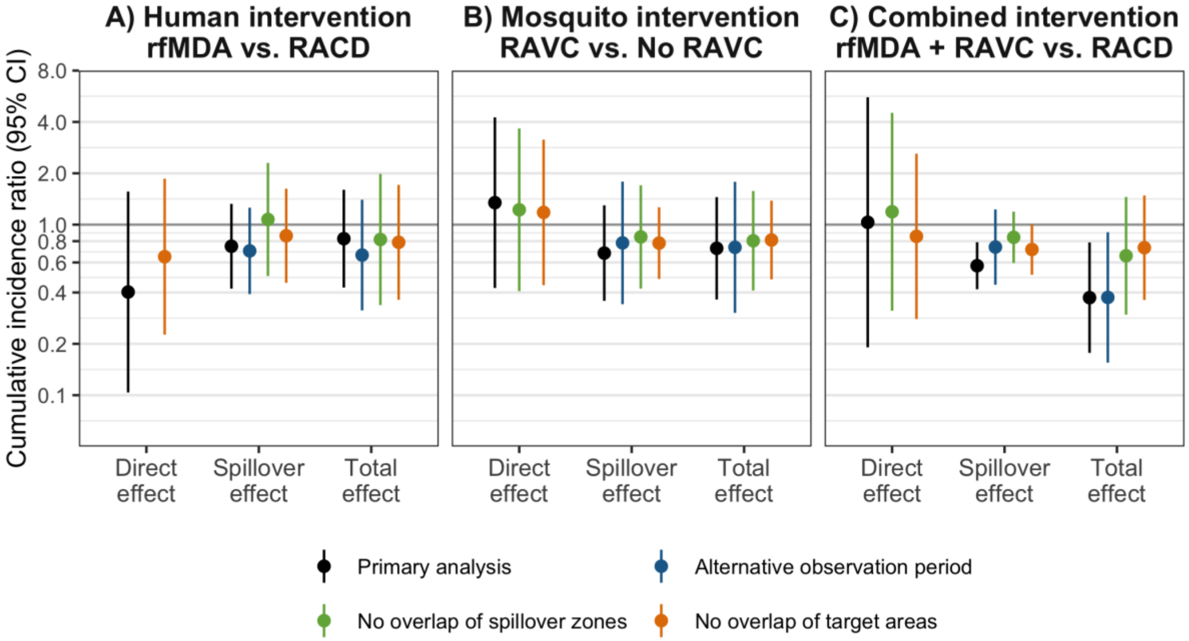

Figure S4. Sensitivity analyses for effects on cumulative incidence of malaria

For rfMDA and RACD arms, the primary analysis includes the period from 0-35 days following index case detection for direct effects and 21-56 days for spillover effects; the alternative observation period analysis includes the period from 0-21 days following index case detection for direct effects and 21 to 42 days for spillover effects. For rfMDA+RAVC and RAVC only arms, the primary analysis includes the period from 0-6 months following index case detection for direct effects and 17 days to 6 months for spillover effects; the alternative observation period analysis includes the period from 0-7 days following index case detection for direct effects and 17 to 90 days for spillover effects. Total effects analyses include the person-time for the direct effects and spillover effects analyses. Direct effect includes intervention recipients in target zone. Spillover effect includes intervention non-recipients up to 1km from an index case. Total effect includes all individuals (intervention recipients and non-recipients) up to 1km from index case. Sensitivity analyses for no overlap of spillover zones excluded any cohorts whose spillover zones overlapped spatially or temporally with other spillover zones. Sensitivity analyses for no overlap of target areas excluded any cohorts whose target areas overlapped spatially or temporally with other target areas. Some direct effects models could not be fit due to data sparsity. All incidence outcome models were fit with cohort-level data except for models of spillover effects of rfMDA vs. RACD and rfMDA + RAVC vs. RACD only.

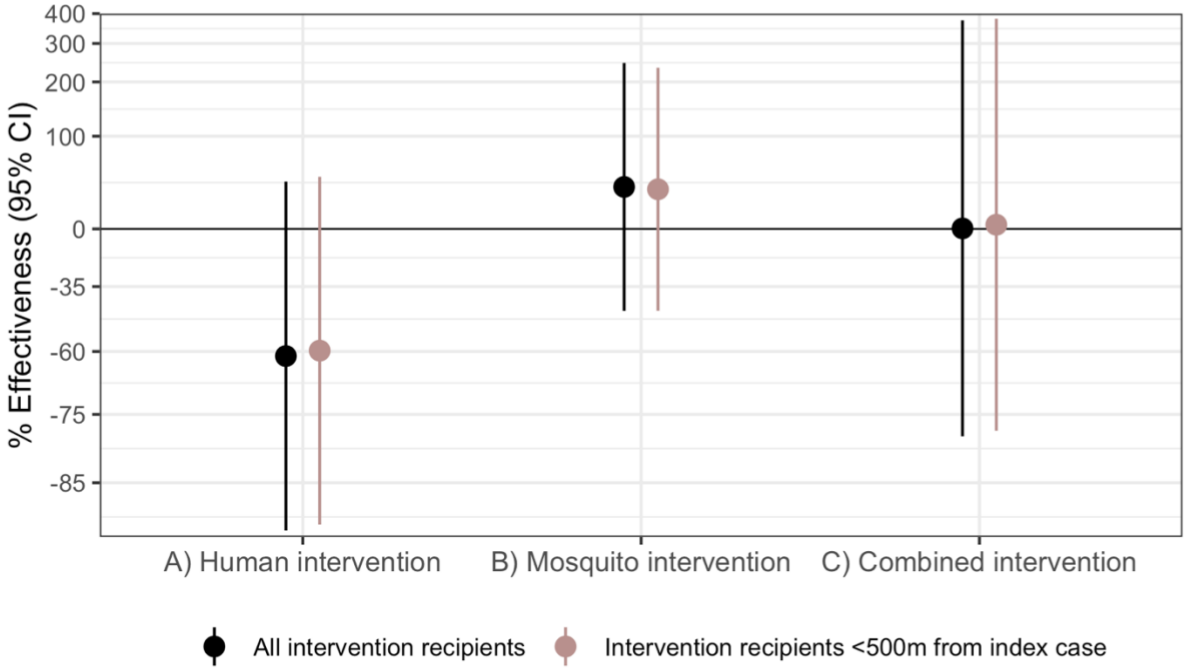

Figure S5. Sensitivity analyses for direct effects including all intervention recipients
The observation period was 0-35 days for rfMDA and RACD arms and 0-6 months for rfMDA+RAVC and RAVC only arms. Black points indicate estimates from analyses including all intervention recipients, regardless of whether they resided within the target zone within 500m of index cases. Mauve points indicate estimates from analyses restricting to intervention recipients within 500m of index cases that triggered interventions. Analyses were performed at the cohort level.

		Human intervention	
		**Reactive case detection only** (28 clusters)	**Reactive focal mass drug administration** (28 clusters)
**Mosquito intervention**	**No reactive focal vector control** (28 clusters)	Reactive case detection only (14 clusters)	Reactive focal mass drug administration only (14 clusters)
	**Reactive focal vector control** (28 clusters)	Reactive case detection plus reactive focal vector control (14 clusters)	Reactive focal mass drug administration plus reactive focal vector control (14 clusters)

Table S1. Two-by-two factorial study design of reactive focal interventions
Reactive case detection (RACD) involved administering rapid diagnostic tests for malaria to individuals living within a 500-m radius of an index case and treating individuals who tested positive with artemether-lumefantrine and single-dose primaquine. Reactive focal mass drug administration (rfMDA) involved presumptively treating individuals living within a 500-m radius of an index case with artemether-lumefantrine, without testing for malaria beforehand. Reactive focal vector control (RAVC) involved spraying the long-lasting insecticide, pirimiphos-methyl, to the interior walls of households located within a seven-household radius of an index case. The effectiveness of three interventions were compared to three respective controls: (1) rfMDA versus RACD (B and D vs A and C); (2) RAVC versus no RAVC (C and D vs A and B); and (3) rfMDA plus RAVC versus a RACD only (D vs A). Reproduced from Hsiang et al. 2020 *Lancet* with permission.

	Human intervention		Mosquito intervention		Human & mosquito intervention	
	RACD	rfMDA	No RAVC	RAVC	RACD only	rfMDA + RAVC
**Population characteristics**						
Number of cohorts	161	149	152	158	73	70
Mean cohort population size (SE)	26 (1)	27 (1)	26 (1)	27 (1)	26 (1)	29 (1)
Mean cluster population size (SE)	389.6 (1.94)	346.4 (1.96)	358.9 (2.01)	376.9 (1.94)	353.0 (2.05)	328.0 (1.89)
Malaria incidence per 1,000 in 2016 (SE)	27.0 (0.37)	55.8 (1.26)	31.9 (0.60)	50.0 (1.15)	26.6 (0.56)	75.4 (2.25)
Pre-season indoor residual spray coverage 2016 (SE)	76.3 (0.32)	77.1 (0.36)	77.9 (0.37)	75.6 (0.31)	83.6 (0.43)	81.5 (0.42)
Distance to nearest healthcare facility (km) (SE)	5.2 (0.06)	6.7 (0.08)	5.0 (0.06)	6.7 (0.07)	3.5 (0.06)	6.9 (0.12)
**Ecological factors (range)**						
Median monthly rainfall November 2016-April 2017 (mm)	23.7 (18.4, 26.7)	23.5 (18.4, 26.7)	23.5 (18.4, 26.7)	23.7 (18.4, 26.7)	23.7 (18.4, 26.7)	23.7 (18.4, 26.7)
Median enhanced vegetative index January 2017-July 2017	0.15 (0.09, 0.31)	0.15 (0.09, 0.27)	0.15 (0.09, 0.22)	0.15 (0.09, 0.31)	0.15 (0.10, 0.21)	0.15 (0.09, 0.27)
Median elevation (m)	522 (387, 1021)	541 (412, 1124)	527 (398, 1124)	547 (387, 1021)	522 (398, 921)	576 (412, 984)
Median daytime land surface temperature (C)	30.5 (28.9, 33.4)	31.1 (28.6, 32.5)	30.7 (28.6, 33.4)	30.8 (28.7, 32.5)	30.7 (28.9, 33.4)	31.1 (28.7, 32.5)

Table S2. Baseline characteristics among intervention recipients

Includes data from intervention recipients in target areas located within 500m of an index case.

	Human intervention		Mosquito intervention		Human & mosquito intervention	
	RACD	rfMDA	No RAVC	RAVC	RACD only	rfMDA + RAVC
**Population characteristics**						
Number of cohorts	161	149	152	158	73	70
Mean cohort population size (SE)	238 (9)	232 (12)	223 (9)	247 (11)	256 (13)	276 (19)
Mean cluster population size (SE)	379.4 (0.63)	355.8 (0.59)	354.0 (0.58)	380.5 (0.63)	349.5 (0.61)	352.8 (0.63)
Malaria incidence per 1,000 in 2016 (SE)	29.2 (0.12)	41.0 (0.35)	28.3 (0.17)	40.4 (0.29)	27.3 (0.16)	50.0 (0.55)
Pre-season indoor residual spray coverage 2016 (SE)	77.1 (0.10)	81.0 (0.12)	78.6 (0.12)	79.2 (0.10)	82.8 (0.14)	86.9 (0.12)
Distance to nearest healthcare facility (km) (SE)	4.9 (0.02)	6.7 (0.03)	4.4 (0.02)	6.9 (0.02)	3.2 (0.02)	7.3 (0.04)
**Ecological factors (range)**						
Median monthly rainfall November 2016-April 2017 (mm)	23.7 (18.4, 26.7)	23.5 (18.4, 26.7)	23.7 (18.4, 26.7)	23.7 (18.4, 26.7)	23.7 (18.4, 26.7)	23.7 (18.4, 26.7)
Median enhanced vegetative index January 2017-July 2017	0.15 (0.09, 0.31)	0.15 (0.09, 0.27)	0.15 (0.09, 0.22)	0.15 (0.09, 0.31)	0.15 (0.10, 0.21)	0.15 (0.09, 0.27)
Median elevation (m)	522 (387, 1021)	535 (412, 1124)	527 (398, 1124)	547 (387, 1021)	522 (398, 921)	677 (412, 984)
Median daytime land surface temperature (C)	30.5 (28.9, 33.4)	31.1 (28.6, 32.5)	30.7 (28.6, 33.4)	30.8 (28.7, 32.5)	30.6 (28.9, 33.4)	31.1 (28.7, 32.5)

Table S3. Baseline characteristics among non-intervention recipients up to 1km away from index cases
Includes data from intervention non-recipients up to 1km from an index case that triggered interventions.

	N cohorts	N	Incidence proportion		Incidence ratio (95% CI)		
			Intervention arm	Reference arm	Unadjusted	Adjusted	Adjusted, CI adjusted for cohort overlap
**Human intervention** (rfMDA vs. RACD)							
Direct effect	310	8,252	3.4	6.5	0.53 (0.25, 1.11)	0.40 (0.11, 1.48)	0.40 (0.10, 1.56)
Spillover effect	310	72,830	9.0	9.9	0.91 (0.60, 1.37)	0.82 (0.52, 1.29)	0.82 (0.44, 1.51)
Total effect	310	81,082	8.4	9.6	0.88 (0.59, 1.31)	0.83 (0.51, 1.35)	0.83 (0.43, 1.60)
**Mosquito intervention** (RAVC vs. no RAVC)							
Direct effect	310	8,252	8.9	7.6	1.17 (0.62, 2.23)	1.35 (0.54, 3.34)	1.35 (0.43, 4.25)
Spillover effect	310	72,830	12.9	18.5	0.69 (0.47, 1.03)	0.68 (0.46, 1.00)	0.68 (0.36, 1.30)
Total effect	310	81,082	12.5	17.4	0.72 (0.49, 1.06)	0.73 (0.49, 1.08)	0.73 (0.36, 1.45)
**Combined intervention** (rfMDA + RAVC vs. RACD only)							
Direct effect	143	3,914	6.4	7.4	0.87 (0.32, 2.41)	1.03 (0.22, 4.81)	1.03 (0.19, 5.58)
Spillover effect	143	38,048	11.2	18.1	0.62 (0.34, 1.13)	0.57 (0.41, 0.80)	0.57 (0.42, 0.79)
Total effect	143	41,962	10.8	17.1	0.63 (0.35, 1.12)	0.37 (0.22, 0.63)	0.37 (0.18, 0.79)

Table S4. Direct effect, spillover effect, and total effect estimates on cumulative incidence of malaria infection

For rfMDA and RACD arms, the analysis includes the period from 0-35 days following index case detection for direct effects and 21-56 days for spillover effects. For rfMDA+RAVC and RAVC only arms, the analysis includes the period from 0-6 months following index case detection for direct effects and 17 days to 6 months for spillover effects. Total effects analyses include the person-time for the direct effects and spillover effects analyses. Direct effect includes intervention recipients in the target zone. Spillover effect analyses includes intervention non-recipients up to 1km from an index case. Total effect includes all individuals (intervention recipients and non-recipients) up to 1km from index case. Models were fit with hierarchical targeted maximum likelihood. All outcome models were fit with cohort-level data except for models of spillover effects of rfMDA + RAVC vs. RACD only. Adjusted models were fit if there were fewer than 10 malaria cases per variable. Covariates were screened separately for each model using a likelihood ratio test. We separately fit individual- and cohort-level outcome models and report the model with the smaller cross-validated mean squared error. All models except spillover effects of the human and combined interventions were fit on cohort-level data.

	Below median		Above median	
	**Minimum**	**Maximum**	**Minimum**	**Maximum**
Malaria incidence per 1,000 in 2016	0.0	13.9	14.9	293.3
Pre-season indoor residual spray coverage 2016 (%)	27.2	77.3	77.9	100
Median daytime land surface temperature (C)	28.6	31.1	31.1	33.4
Median monthly rainfall November 2016-April 2017 (mm)	18.4	23.7	23.7	26.7
Median enhanced vegetative index January 2017-July 2017	0.09	0.15	0.15	0.31
Median elevation (m)	387	541	544	1124
Cohort-level treatment coverage (%)	0.0	8.3	8.3	97.4

Table S5. Range above and below median value in each enumeration area for subgroup variables

	Primary analysis		Sensitivity analysis with shorter observation period	
	**Target areas**	**Spillover zone**	**Target areas**	**Spillover zone**
**Human intervention** (rfMDA vs. RACD)	32.0	28.9	21.2	18.4
**Mosquito intervention** (RAVC vs. no RAVC)	59.2	47.5	53.8	41.8
**Combined intervention** (rfMDA + RAVC vs. RACD only)	60.5	28.1	60.2	24.1

Table S6. Percentage of cohorts overlapping with other cohorts
Overlap in target area was defined as index cases that triggered interventions located within <1km of each other and observation periods that temporally overlapped with another cohort's. Overlap in spillover zones was defined as index cases that triggered interventions located within 1-2km of each other and observation periods that temporally overlapped with another cohort's. The denominator was the total cohorts included in each analysis.

	N		Prevalence		Prevalence ratio (95% CI)	
	Intervention arm	Reference arm	Intervention arm	Reference arm	Unadjusted	Adjusted
**Human intervention** (rfMDA vs. RACD)						
Direct effect	1537	1835	0.029	0.033	0.90 (0.61, 1.31)	0.84 (0.53, 1.32)
Spillover effect	244	229	0.025	0.087	0.28 (0.12, 0.69)	--
Total effect	1781	2064	0.029	0.039	0.74 (0.52, 1.04)	0.79 (0.51, 1.19)
**Mosquito intervention** (RAVC vs. no RAVC)						
Direct effect	1710	1662	0.026	0.037	0.70 (0.48, 1.03)	0.78 (0.51, 1.21)
Spillover effect	195	278	0.051	0.058	0.89 (0.41, 1.92)	--
Total effect	1905	1940	0.028	0.040	0.71 (0.51, 1.01)	0.64 (0.43, 0.96)
**Combined intervention** (rfMDA + RAVC vs. RACD only)						
Direct effect	758	883	0.017	0.033	0.52 (0.27, 1.00)	--
Spillover effect	118	152	0.017	0.079	0.21 (0.05, 0.94)	--
Total effect	876	1035	0.017	0.040	0.43 (0.24, 0.78)	--

Table S7. Direct effect, spillover effect, and total effect estimates on malaria prevalence measured by qPCR
Prevalence was measured in a cross-sectional survey in a random sample of households at the end of the malaria season. Analyses were restricted to individuals located within 3 km of at least one intervention recipient. Direct effects include individuals with any intervention recipients within 500m, spillover effects include individuals with no intervention recipients < 500m and any intervention recipients 500m-3km, and total effects include individuals with any intervention recipients <3km during the study. Prevalence ratios were estimated using TMLE with individual-level data, and standard errors were adjusted for clustering at the enumeration area level. Adjusted analyses were not fit there were fewer than 30 observations within strata of the intervention and outcome. Adjusted models were not fit if the number of cases within treatment arm strata was <30.

	N households		Prevalence		Unadjusted Prevalence Ratio (95% CI)
	Intervention arm	Reference arm	Intervention arm	Reference arm	
**Human intervention** (rfMDA vs. RACD)					
Direct effect	456	506	0.018	0.018	0.99 (0.38, 2.54)
Spillover effect	72	69	0.000	0.043	0.00 (0.00, 0.00)
Total effect	528	575	0.015	0.021	0.73 (0.30, 1.76)
**Mosquito intervention** (RAVC vs. no RAVC)					
Direct effect	481	481	0.012	0.023	0.55 (0.20, 1.46)
Spillover effect	65	76	0.015	0.026	0.58 (0.05, 6.35)
Total effect	546	557	0.013	0.023	0.55 (0.22, 1.37)
**Combined intervention** (rfMDA + RAVC vs. RACD only)					
Direct effect	219	244	0.005	0.016	0.28 (0.03, 2.48)
Spillover effect	36	40	0.000	0.050	0.00 (0.00, 0.00)
Total effect	255	284	0.004	0.021	0.19 (0.02, 1.53)

Table S8. Direct effect, spillover effect, and total effect estimates on household-level malaria prevalence of measured by qPCR
Prevalence was measured in a cross-sectional survey in a random sample of households at the end of the malaria season. Analyses were run at the household level. Household-level malaria prevalence was the percentage of households with more than one malaria case detected in the prevalence survey by qPCR. Direct effects include households with any intervention recipients within 500m, spillover effects include households with no intervention recipients < 500m and any intervention recipients 500m-3km, and total effects include households with any intervention recipients <3km during the study. Prevalence ratios were estimated using TMLE with household-level data. Adjusted analyses were not fit there were fewer than 30 observations within strata of the intervention and outcome. Adjusted models were not fit if the number of cases within treatment arm strata was <30.

	N		Prevalence		Prevalence ratio (95% CI)	
	Intervention arm	Reference arm	Intervention arm	Reference arm	Unadjusted	Adjusted
**Human intervention** (rfMDA vs. RACD)						
Direct effect	1316	1611	0.215	0.285	0.75 (0.66, 0.86)	0.84 (0.71, 1.00)
Spillover effect	198	182	0.227	0.225	1.01 (0.69, 1.46)	1.32 (0.73, 2.41)
Total effect	1514	1793	0.217	0.279	0.78 (0.69, 0.88)	0.85 (0.73, 0.99)
**Mosquito intervention** (RAVC vs. no RAVC)						
Direct effect	1475	1452	0.241	0.267	0.90 (0.80, 1.02)	0.90 (0.79, 1.04)
Spillover effect	133	247	0.188	0.247	0.76 (0.50, 1.15)	--
Total effect	1608	1699	0.236	0.264	0.90 (0.80, 1.01)	0.88 (0.76, 1.01)
**Combined intervention** (rfMDA + RAVC vs. RACD only)						
Direct effect	634	770	0.194	0.295	0.66 (0.54, 0.80)	--
Spillover effect	81	130	0.136	0.208	0.66 (0.55, 0.80)	--
Total effect	715	900	0.187	0.282	0.65 (0.34, 1.25)	--

Table S9. Direct effect, spillover effect, and total effect estimates on Etramp5.Ag1 seroprevalence
Prevalence was measured in a cross-sectional survey in a random sample of households at the end of the malaria season. Analyses were restricted to individuals located within 3 km of at least one intervention recipient. Direct effects include individuals with any intervention recipients within 500m, spillover effects include individuals with no intervention recipients < 500m and any intervention recipients 500m-3km, and total effects include individuals with any intervention recipients <3km during the study. Prevalence ratios were estimated using TMLE with individual-level data, and standard errors were adjusted for clustering at the enumeration area level. Adjusted analyses were not fit there were fewer than 30 observations within strata of the intervention and outcome. Adjusted models were not fit if the number of cases within treatment arm strata was <30.

		N individuals		Prevalence		Prevalent cases		Total prevalent cases averted (95% CI)	Incremental cost-effectiveness ratio (95% CI)	% change from original estimate
	Intervention cost	Target area	Spillover zone	Target area	Spillover zone	Target area	Spillover zone			
**Human intervention**										
RACD	$354,750	8,187	996	0.033	0.087	268	87	(ref)	(ref)	
rfMDA	$368,321	8,060	1,301	0.029	0.025	236	32	87 (77, 96)	$156 ($141, $177)	-3%
**Mosquito intervention**										
No RAVC	$261,409	7,845	1,290	0.037	0.058	288	74	(ref)	(ref)	
RAVC	$461,661	8,426	980	0.026	0.051	217	50	95 (82, 108)	$2,105 ($1,859, $2,430)	-21%
**Combined intervention**										
RACD only	$127,312	3,697	626	0.033	0.079	121	49	(ref)	(ref)	
rfMDA+RAVC	$234,223	3,878	635	0.017	0.017	66	11	94 (74, 113)	$1,142 ($944, $1,446)	-37%

Table S10. Cost-effectiveness analysis

Prevalent cases averted were estimated using hierarchical TMLE models for prevalence measured by qPCR. The number of prevalent cases averted equaled the produce of the difference in prevalence between arms among intervention recipients and non-recipients by the estimated population size within target areas vs. spillover zones. The incremental cost effectiveness ratio is the ratio of the difference in cost between arms by the difference in prevalent cases averted in both target area and spillover zones within 3 km of index cases for rfMDA + RAVC vs. RACD. Original estimates were reported in Ntuku et al., 2022 10.1136/bmjopen-2021-049050.

medRxiv preprint doi: https://doi.org/10.1101/2023.09.19.23295806; this version posted November 30, 2023. The copyright holder for this preprint (which was not certified by peer review) is the author/funder, who has granted medRxiv a license to display the preprint in perpetuity.

It is made available under a CC-BY 4.0 International license .

Supporting Information

Study population

This study analyzed data from a cluster-randomized trial of focal malaria interventions conducted in Zambezi region of Namibia from January 1 to December 31, 2017 (NCT02610400) (1, 2). The region has seasonal malaria transmission that peaks between January and June. *Plasmodium falciparum* is the dominant species, and annual *Pf* incidence was less than 15 per 1,000 from 2010-2015. In 2016, the incidence was 32.5 per 1,000 following an outbreak (3). In 2015, prevalence measured by loop-mediated isothermal amplification was 2.2% (4). In the study site, the Namibia Ministry of Health and Social Services routinely delivered case management and annual preseason household IRS with dichlorodiphenyltrichloroethane, with the exception of a small number of structures that were sprayed with deltamethrin. In addition, they offered reactive case detection (RACD) within 500 m of confirmed malaria cases, which included testing with rapid diagnostic tests and treatment with artemether-lumefrantrine and single-dose primaquine for those who tested positive.

Cluster-randomized trial design

The trial included 56 clusters defined based on census enumeration areas that were within the catchment area of study health care facilities. Enumeration areas were eligible for inclusion in the trial if they 1) were located in the catchment areas of 11 health facilities, 2) had complete incidence data from 2012-13, and 3) had at least one incident case during the trial. Using a two-by-two factorial design, the trial randomized 56 clusters to four arms: 1) RACD only, 2) reactive focal mass drug administration (rfMDA) only, 3) reactive vector control (RAVC) + RACD, 4) RAVC + rfMDA. rfMDA included presumptive treatment with artemether-lumefrantrine to individuals in target areas (Extended Data Table 1). The trial used restricted randomization with the following criteria: mean annual incidence in 2013 and 2014, population size, population density, and mean distance from the household to a health-care facility. It was not practical to blind study participants or field staff to intervention assignment, but laboratory analyses and primary statistical analyses were blinded.

Interventions

Field staff delivered interventions in response to passively detected malaria index cases that were confirmed by rapid diagnostic tests or microscopy if the case had resided in the study cluster at least one night in the prior 4 weeks. The trial delivered interventions in “target areas” within approximately 500 m of confirmed malaria cases detected through passive surveillance. In the RACD arms, individuals were eligible to receive rapid diagnostic tests, and individuals who tested positive were eligible for treatment with artemether-lumefrantrine and single-dose primaquine (Coartem, Novartis Pharmaceuticals, Kempton Park, South Africa; or Komefan 140, Mylan Laboratories, Sinnar, India). In the rfMDA arms, individuals were eligible for presumptive treatment with artemether-lumefrantrine. In the RAVC arms, households were eligible for IRS with pirimiphosmethyl (Actellic 300CS, Syngenta, Basel, Switzerland). In all arms, study teams aimed to deliver interventions within 500 m of a clinical malaria case and within 7 days to 5 weeks of the case report. RACD and rfMDA interventions were delivered to at least 25 people within target areas and RAVC was delivered to at least seven households within target areas.

medRxiv preprint doi: https://doi.org/10.1101/2023.09.19.23295806; this version posted November 30, 2023. The copyright holder for this preprint (which was not certified by peer review) is the author/funder, who has granted medRxiv a license to display the preprint in perpetuity.

It is made available under a CC-BY 4.0 International license .

Over 80% of eligible confirmed malaria cases received interventions, and over 85% of eligible intervention recipients were covered by interventions (2). Since compliance was high, for intervention recipients, we analyzed treatment as randomly assigned. Field staff did not offer repeat interventions in response to subsequent index cases within 5 weeks for rfMDA and RACD and within the same malaria season for RAVC. Field staff recorded the household geocoordinates of the index case and intervention recipients. Additional details about the interventions were previously published (1, 2).

Procedures

Prior to randomization, field staff conducted a geographic census and recorded the latitude and longitude of all households in the study area. During the trial, trial staff extracted data on confirmed incident malaria cases and travel history from the rapid reporting system. At the end of malaria season between May and August 2017, the study team collected an endline cross-sectional survey to measure infection prevalence. Field staff collected dried blood spots on filter paper (Whatman 3 Corporation, Florham Park, NJ, USA) by finger prick from consenting individuals, and qPCR was performed targeting the acidic terminal sequence of the *var* gene.(5) Field staff also collected 250 ml of whole blood in BD Microtainer tubes with EDTA additive (Becton, Dickinson and Corporation, Franklin Lakes, NJ, USA) for serological analyses. Using human plasma, Luminex assays were performed to detect malaria antigens using previously described procedures (6, 7). Field staff recorded the geocoordinates of all sampled households.

Informed consent

In the original trial, written informed consent was obtained from individual participants for rfMDA or RACD, and from heads of households (≥18 years of age) for RAVC. A parent or guardian was required to provide written informed consent for children younger than 18 years receiving rfMDA or RACD, and written assent for receiving these interventions was also obtained from children aged 12–17 years.

Construction of analytic cohorts for incidence analysis

To construct cohorts, we matched index cases and intervention recipients to individuals recorded in the baseline census using household geocoordinates, age, and sex. We required that geocoordinates be < 100m apart to allow for small deviations in the location of geocoordinate recordings. We excluded 32 cohorts from the analysis for which it was not possible to merge intervention recipient geocoordinates with index data geocoordinates. Because clusters were contiguous with no buffer zones between them, to capture potential dependencies across study clusters, we allowed cohorts to include individuals assigned to an adjacent cluster with a different treatment assignment from the triggering index case if it was within 1 km of an index case.

Follow-up periods for analytic cohorts

We pre-specified cohort follow-up length based on the period in which we expected each intervention to reduce malaria among intervention recipients (direct effects) and non-recipients (spillover effects). Day 0 for each cohort was the date of index case detection. For comparisons of rfMDA and RACD interventions, the direct effect follow-up period was 0 to 35 days, the

medRxiv preprint doi: https://doi.org/10.1101/2023.09.19.23295806; this version posted November 30, 2023. The copyright holder for this preprint (which was not certified by peer review) is the author/funder, who has granted medRxiv a license to display the preprint in perpetuity.
It is made available under a CC-BY 4.0 International license .

length of intrinsic incubation period for *Pf* malaria (8). This is the period of time in which we would expect the intervention to interrupt the parasite life cycle in treated, infected individuals, and in turn, prevent symptoms and/or infectiousness. The spillover effect follow-up period was 21 to 56 days; the 3-week lag period allowed for gametocyte clearance in the treated individual, sporozoite development in mosquitos, and development of detectable merozoites in humans. For RAVC interventions, the direct effects follow-up period was 6 months since IRS can remain effective for an entire transmission season (9). The spillover effects follow-up period was from day 17 to 6 months. A mosquito bite could hypothetically be prevented on the day of intervention, so the earliest secondary case could occur after sporozoite development in mosquitos (minimum 10 days), and development of detectable merozoites in humans (minimum 7 days). We conducted a sensitivity analysis with alternative follow-up lengths (rfMDA and RACD direct effects: day 0-21; spillover effects: day 21-42; RAVC direct effects day 0-7; spillover effects day 17-90).

Hierarchical TMLE

We compared incidence between arms using hierarchical targeted maximum likelihood estimation (TMLE) (10). We fit propensity score models at the cohort-level since interventions were delivered to cohorts. Within study clusters and cohorts, we expected individuals' outcomes to be correlated due to interventions, social interactions, and local environmental factors. We fit two types of outcome models that accounted for statistical dependence in different ways (11). Cohort-level models allowed for statistical dependence between individuals in the same cohort without making any assumptions about the nature of the dependency. Individual-level models assumed that cluster-level and individual-level covariates removed any dependence between outcomes of individuals in nearby geographic areas (11). We separately fit individual- and cohort-level models and then chose the outcome model with the smaller cross-validated mean squared error.

We fit outcome and propensity score models using an ensemble machine learning algorithm (the Superlearner) (12). For propensity score models, learners included generalized linear models, least absolute shrinkage and selection operator (LASSO) (13), and elastic net regression (14). For outcome models, we used the same learners as well as extreme gradient boosting (15). We performed 10-fold cross-validation using a loss function at either the individual- or cohort-level (11). Validation samples were constructed from randomly sampled individuals or cohorts. Because comparisons of rfMDA + RAVC vs. RACD had rare outcomes and a smaller sample size, we used 30-fold cross-validation.

Adjusting standard errors for cohort overlap

We adjusted standard errors to account for potential correlation due to overlap between some cohorts using a model of cohort-level influence curves analogous to variance-covariance models used in cross-random effects models (16, 17). Specifically, we fit the model:

$$D_i \times D_j \sim d(i,j) + t(i,j) + C \qquad (1)$$

medRxiv preprint doi: https://doi.org/10.1101/2023.09.19.23295806; this version posted November 30, 2023. The copyright holder for this preprint (which was not certified by peer review) is the author/funder, who has granted medRxiv a license to display the preprint in perpetuity.

It is made available under a CC-BY 4.0 International license .

where $D_i \times D_j$ is the product of influence curves of cohorts *i* and *j*, $d(i,j)$ is the distance between the location of the index case that triggered the intervention in each cohort, $t(i,j)$ is the start date of the intervention in each cohort, and *C* is the cluster-level intervention assignment (18). Adjustment for intervention assignment accounted for correlation due to shared exposure to or receipt of the intervention. For cohorts with no overlap, we set $D_i \times D_j$ to zero. The regression was implemented with a simplified SuperLearner library including the generalized linear models and LASSO (13). We calculated the variance accounting for outcome dependence as follows:

$$var(\hat{\psi} - \psi) = var\left(\frac{1}{N}\sum_{i=1}^{N} D_i\right) = \frac{1}{N^2}\ (\sum_{i=1}^{N} var(D_i)\ +\ 2\sum_{i<j} cov(D_i, D_j))$$

where $\hat{\psi}$ is the estimator, ψ is the estimand, and N is the number of cohorts.

In both incidence and prevalence analyses, we excluded any categorical covariates with less than 5% prevalence to avoid positivity violations. To minimize empirical positivity violations (19), we only fit models if the number of outcome events per variable was ≥10 and only fit adjusted models if the number of observations per strata was ≥30 (20).

medRxiv preprint doi: https://doi.org/10.1101/2023.09.19.23295806; this version posted November 30, 2023. The copyright holder for this preprint (which was not certified by peer review) is the author/funder, who has granted medRxiv a license to display the preprint in perpetuity.

It is made available under a CC-BY 4.0 International license .

Deviations from pre-analysis plan

The analysis plan for this study was pre-specified at https://osf.io/s8ay4/. We note the following deviations from the plan:

1. We originally planned to conduct an individual participant data meta-analysis including data from three trials in Namibia, Eswatini, and Zambia. However, after reviewing the data for the Eswatini and Zambia trials, we determined that the geocoding of participants was not sufficient to allow for the planned spillover analyses. Thus, we proceeded with an analysis using data only from the Namibia trial.

2. In primary analyses using incidence data, we did not impose bounds on the mean outcome conditional on treatment and covariates because in initial models using bounds, estimates were very unstable.

3. In secondary analyses using prevalence data, we corrected standard errors at the cluster-level instead of at the household-level as specified in the pre-analysis plan. This better reflected the clustered sampling in the original trial.

medRxiv preprint doi: https://doi.org/10.1101/2023.09.19.23295806; this version posted November 30, 2023. The copyright holder for this preprint (which was not certified by peer review) is the author/funder, who has granted medRxiv a license to display the preprint in perpetuity.

It is made available under a CC-BY 4.0 International license .

References

1. O. F. Medzihradsky, *et al.*, Study protocol for a cluster randomised controlled factorial design trial to assess the effectiveness and feasibility of reactive focal mass drug administration and vector control to reduce malaria transmission in the low endemic setting of Namibia. *BMJ Open* **8** (2018).

2. M. S. Hsiang, *et al.*, The effectiveness of reactive focal mass drug administration (rfMDA) and reactive focal vector control (RAVC) to reduce malaria transmission: a cluster-randomised controlled open label two-by-two factorial design trial from the low-endemic setting of Namibia. **395**, 1361–1373 (2020).

3. E. Chanda, *et al.*, An investigation of the Plasmodium falciparum malaria epidemic in Kavango and Zambezi regions of Namibia in 2016. *Transactions of The Royal Society of Tropical Medicine and Hygiene* **112**, 546–554 (2018).

4. P. McCreesh, *et al.*, Subpatent malaria in a low transmission African setting: a cross-sectional study using rapid diagnostic testing (RDT) and loop-mediated isothermal amplification (LAMP) from Zambezi region, Namibia. *Malar J* **17**, 480 (2018).

5. N. Hofmann, *et al.*, Ultra-Sensitive Detection of Plasmodium falciparum by Amplification of Multi-Copy Subtelomeric Targets. *PLOS Medicine* **12**, e1001788 (2015).

6. L. Wu, *et al.*, Optimisation and standardisation of a multiplex immunoassay of diverse Plasmodium falciparum antigens to assess changes in malaria transmission using sero-epidemiology. *Wellcome Open Res* **4**, 26 (2020).

7. L. Wu, *et al.*, Serological evaluation of the effectiveness of reactive focal mass drug administration and reactive vector control to reduce malaria transmission in Zambezi Region, Namibia: Results from a secondary analysis of a cluster randomised trial. *eClinicalMedicine* **44**, 101272 (2022).

8. S. K. Nilsson, L. M. Childs, C. Buckee, M. Marti, Targeting Human Transmission Biology for Malaria Elimination. *PLOS Pathogens* **11**, e1004871 (2015).

9. World Health Organization, "WHO recommended insecticides for indoor residual spraying against malaria vectors" (2018).

10. W. Zheng, M. J. van der Laan, "Cross-Validated Targeted Minimum-Loss-Based Estimation" in *Targeted Learning: Causal Inference for Observational and Experimental Data*, Springer Series in Statistics., M. J. van der Laan, S. Rose, Eds. (Springer, 2011), pp. 459–474.

11. L. B. Balzer, W. Zheng, M. J. van der Laan, M. L. Petersen, A new approach to hierarchical data analysis: Targeted maximum likelihood estimation for the causal effect of a cluster-level exposure. *Stat Methods Med Res* **28**, 1761–1780 (2019).

12. M. J. van der Laan, E. C. Polley, A. E. Hubbard, Super learner. *Stat Appl Genet Mol Biol* **6**, Article 25 (2007).

13. R. Tibshirani, Regression Shrinkage and Selection via the Lasso. *Journal of the Royal Statistical Society. Series B (Methodological)* **58**, 267–288 (1996).

medRxiv preprint doi: https://doi.org/10.1101/2023.09.19.23295806; this version posted November 30, 2023. The copyright holder for this preprint (which was not certified by peer review) is the author/funder, who has granted medRxiv a license to display the preprint in perpetuity.

It is made available under a CC-BY 4.0 International license .

14. J. Friedman, T. Hastie, R. Tibshirani, Regularization Paths for Generalized Linear Models via Coordinate Descent. *J Stat Softw* **33**, 1–22 (2010).

15. J. H. Friedman, Greedy Function Approximation: A Gradient Boosting Machine. *The Annals of Statistics* **29**, 1189–1232 (2001).

16. H. Goldstein, Multilevel Covariance Component Models. *Biometrika* **74**, 430–431 (1987).

17. S. W. Raudenbush, A Crossed Random Effects Model for Unbalanced Data With Applications in Cross-Sectional and Longitudinal Research. *Journal of Educational Statistics* **18**, 321–349 (1993).

18. M. M. Davies, M. J. van der Laan, Sieve Plateau Variance Estimators: A New Approach to Confidence Interval Estimation for Dependent Data. *U.C. Berkeley Division of Biostatistics Working Paper Series* **Working Paper 322** (2014).

19. M. L. Petersen, K. E. Porter, S. Gruber, Y. Wang, M. J. van der Laan, Diagnosing and responding to violations in the positivity assumption. *Stat Methods Med Res* **21**, 31–54 (2010).

20. P. Peduzzi, J. Concato, E. Kemper, T. R. Holford, A. R. Feinstein, A simulation study of the number of events per variable in logistic regression analysis. *Journal of Clinical Epidemiology* **49**, 1373–1379 (1996).
